# Notable improvements on LWFA through precise laser wavefront tuning

**DOI:** 10.1038/s41598-023-45737-5

**Published:** 2023-10-27

**Authors:** Driss Oumbarek Espinos, Alexandre Rondepierre, Alexei Zhidkov, Naveen Pathak, Zhan Jin, Kai Huang, Nobuhiko Nakanii, Izuru Daito, Masaki Kando, Tomonao Hosokai

**Affiliations:** 1 Institute of Scientific and Industrial Research (SANKEN), Osaka University, Mihogaoka, Ibaraki, Osaka 565-0871 Japan; 2 Laser Accelerator R &D, Innovative Light Sources Division, RIKEN SPring-8 Center, 1-1-1, Kouto, Sayo-cho, Sayo-gun, Hyogo, Osaka 679-5148 Japan; 3 Kansai Institute for Photon Science (KPSI), National Institutes for Quantum Science and Technology (QST), 8-1-7, Umemidai, Kizugawa, Kyoto 619-0215 Japan

**Keywords:** Laser-produced plasmas, Plasma-based accelerators

## Abstract

Laser wakefield acceleration (LWFA) continues to grow and awaken interest worldwide, especially as in various applications it approaches performance comparable to classical accelerators. However, numerous challenges still exist until this can be a reality. The complex non-linear nature of the process of interaction between the laser and the induced plasma remains an obstacle to the widespread LWFA use as stable and reliable particle sources. It is commonly accepted that the best wavefront is a perfect Gaussian distribution. However, experimentally, this is not correct and more complicated ones can potentially give better results. in this work, the effects of tuning the laser wavefront via the controlled introduction of aberrations are explored for an LWFA accelerator using the shock injection configuration. Our experiments show the clear unique correlation between the generated beam transverse characteristics and the different input wavefronts. The electron beams stability, acceleration and injection are also significantly different. We found that in our case, the best beams were generated with a specific complex wavefront. A greater understanding of electron generation as function of the laser input is achieved thanks to this method and hopes towards a higher level of control on the electrons beams by LWFA is foreseen.

## Introduction

Since the conception of laser wakefield acceleration (LWFA)^1^ this technique has vastly advanced, to the point that its use as electron beam source for free electron laser^2^, notorious for its strict requirements, has been already achieved^3,4^, paving the way to more future uses. In LWFA, a high intensity fs laser propagates inside a gas, ionizing it and expelling the plasma electrons from its path via the ponderomotive force. An electronless area (wake) is created behind the laser in which acceleration gradients of up to hundreds of GV/m can be achieved. The high degree of non-linearity in the components of a LWFA complicates the control, and even the capacity to understand which conditions to aim, to obtain electron beams capable to equal and surpass the ones of classic accelerators. Simplifying such system to its core components leaves two main non-trivial parts, i.e., the laser system and the gas target.

Regarding the target, its selection is determined by the kind of used LWFA technique, e.g. ionization injection^5^ can be done with a flat gas distribution. In addition, mixed gases^6^ substantially improve (main gas + doping) the technique when in the right proportions. Colliding pulse^7^ needs a careful calculation of the space occupied by the gas and its uniformity to better control both lasers path and interaction time. Shock injection^8,9^ depends critically on the shock parameters (shock density, position, etc) and the following ramp as well as the laser focusing position to assure a controlled and localized injection^10^. Thanks to its controlled injection in the wake (low energy spread), one can separate it from the acceleration part without as much complexity as the colliding scheme.

In all cases, the driver of the LWFA process is the laser pulse. The interaction of a high intensity perfect laser, e.g., Gaussian, with a mm length plasma is already not a simple process, and therefore, taking into account a realistic laser beam, which can be quite far from the perfect Gaussian case regarding its phase distribution (aberrations^11^), its beam quality factor (M$^2$) and hence its near and far field intensity pattern can substantially change the laser-plasma interaction. The inclusion of aberrations and real near field patterns (often a non-homogeneous flat top distribution) greatly affects the laser intensity distribution along its propagation^11^, crucial in the wakefield creation and dynamics. On top of this, the multiple processes occurring inside the plasma (etching^12^, self-focusing^13^, filamentation^14^, etc) originates a highly complex evolution of the laser propagation compared to vacuum conditions. Therefore, in experiment, where laser and gas are not perfect, the LWFA process shows some shot-to-shot instabilities and also puts into question the superiority of aiming for a perfect Gaussian laser pulse when interacting with an imperfect gas target as already hinted in some works^11,15–17^. Beaurepaire et al.^16^ presents a qualitative comparison between two simple laser transverse distributions, reconstructed from experiment, with real and flat wavefront. However, the conclusion is that the wavefront is important for LPA but lacks any deeper study. Similarly, Ferri et al.^18^ measure their non-Gaussian transverse laser wavefront and phase. Through simulations they show that using an initial Gaussian or a non-Gaussian wavefront give up to a 23% difference in the number of photons emitted by the plasma. Nevertheless, the simulations do not take into account a full characterization of the laser pulse, thus making its longitudinal evolution incorrect, and from this work the authors conclude that “improving the laser spot quality would also lead to an important benefit”, thus coming back to the belief that every situation could be improved with a Gaussian ideal laser beam. Lin et al.^19^ through a semi-random optimization (genetic algorithm) applied to a deformable mirror and a change in mid-IR laser focus position finds mainly the possibility of increasing the charge of the low energy electrons (1–4 MeV) however, the same inaccuracy on the simulations is committed and the LPA configuration is quite different (low energy, huge divergence, high charge) from the other works, including ours. Another thing in common in these works is the use of the ionization injection scheme without gas density tailoring.

In this work, we show how by using a complex understood wavefront intensity distribution, defined by the Zernike polynomial terms, a higher quality electron beam has been achieved with respect to the “no aberrations” case (which is the usual target) and a possibility of simultaneous multiple electron beam generation. First, we present the effects on the LWFA electron beam generation of tuning the laser pulse transverse intensity distribution in the vicinity of the waist position by adding aberrations in a controlled fashion. We demonstrate the capabilities to change and even improve the beam characteristics without altering anything on the target side. Furthermore, we explore the distinctive electron beam patterns observed related to different aberration configurations.

## Results

### Configuration

**Figure 1:**
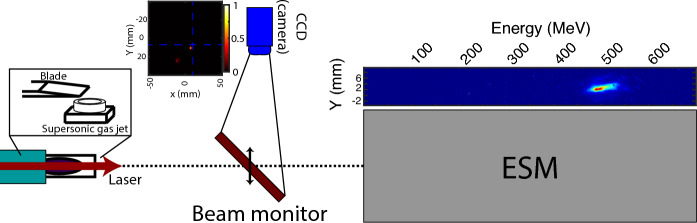
Scheme of the experimental setup with examples of the observed beam monitor measurement and electron spectrometer.

For this experiment, the second beam line of the LAPLACIAN (Laser Acceleration Platform as a Coordinated Innovative Anchor) facility, located at RIKEN SPring-8 Center, was used. For the LWFA, a 800 nm, 23 fs full width half maximum (FWHM) laser with 0.7 J on target energy interacts with a pure He gas target under vacuum. The laser aberrations are modified by a deformable mirror that uses the measurements of a wavefront sensor as feedback. A F/20 parabola focuses the laser onto the gas target with a waist diameter of 20 $\upmu m$. The gas target is prepared in the shock injection configuration as seen in Fig. 1, with a 4 mm long conical supersonic gas jet 4.5 mm under the laser axis and a simple blade 3.5 mm above the jet and with $\approx$ 20% coverage (percentage of the gas distribution covered by the blade in the longitudinal direction^20^). The gas density is set to around $2 \times 10^{18}$ ${\text{cm}}^{-3}$. The generated electron beams transverse distribution and relative charge are observed in a beam monitor 955 mm after the gas jet and their energy and charge on an electron spectrometer positioned 2 m after the gas jet.

### Concept

**Figure 2:**
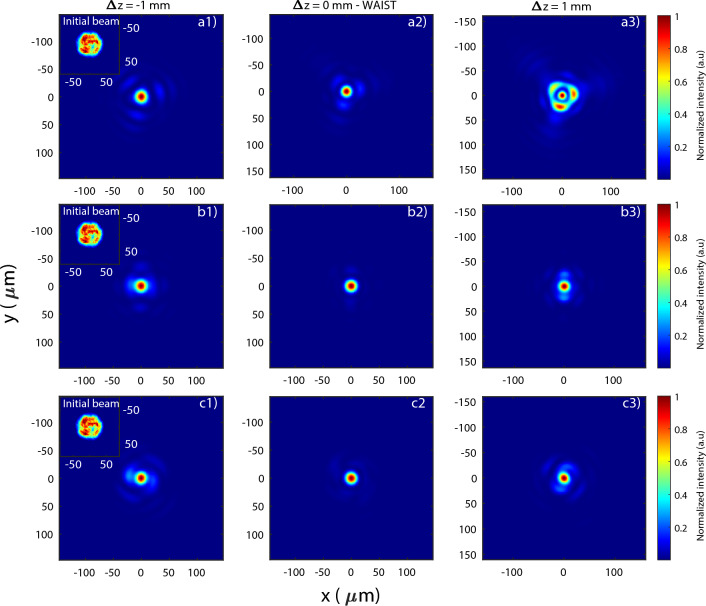
Calculated transverse intensity distribution at focus and $\pm \,1$ mm (see the insert for the near field intensity distribution) when introducing an additional (**a**) $\lambda$/20 of both trefoil 0 and 45, (**b**) $\lambda$/20 of first order astigmatism 0 and (**c**) $\lambda$/14 of second order astigmatism 0.

When a laser pulse is focused, the pattern evolution from its origin up to the focal plane depends on the amplitude and phase patterns. For a simple Gaussian beam free of aberrations, the beam is focused without any disturbance and will remain Gaussian at every position. However, when either a phase is added or the initial profile is differing from the Gaussian one, the beam pattern will become more complex^11^. Figure 2 shows the beam intensity pattern 2 mm around the focal plane during propagation in-vacuum when using the experimental laser: intensity pattern at LAPLACIAN with a flat phase (obtained after correction with a Strehl Ratio of 0.92), and considering an additional phase error. The added phase were $\lambda$/20 of both trefoil 0 and 45 (Fig. 2a), $\lambda$/20 of first order astigmatism 0 (Fig. 2b) and $\lambda$/14 of second order astigmatism 0 (Fig. 2c). The phase error is given in RMS over the whole pupil analyzed in $\lambda$ units (an error of $\lambda$ stands for an error of $2\pi$ radians). The focus position was defined as the position where the beam size is the smallest without aberration (reference) and this position was kept the same (until otherwise is specified) when applying others configurations. However, what is called the best focus position may change compared to the reference one. Adding a gas starting around $\approx 1.5$ mm before the focus position further changes the laser intensity distribution due to the multiple non-linear effects, e.g., filamentation, etching, self-focusing. Furthermore, experimentally the gas distribution is not as perfect as in simulations. Previous works have already shown the importance of the up and down-ramps of the gas distributions and of the differences between a middle region closer to a flat-top or Gaussian distribution^21^.

**Figure 3:**
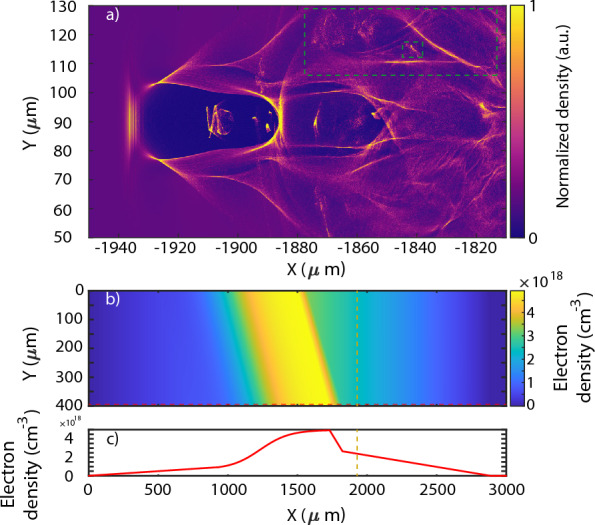
(**a**) Laser wakefield acceleration 2D PIC simulation done with FPlaser (laser propagation to the left) after 1.94 mm of propagation (mustard dotted line). The green squares indicate the secondary particle driven wakefield with some electrons injected. (**b**) Used 2D density distribution and bottom (red dotted line). (**c**) 1D distribution (propagation direction to the right).

In the specific case of the LWFA shock configuration one should also take care of the shock position and its angle with respect to the laser axis. In Dr. Tsai’s work^22^ it is shown how straightening the shock to achieve a quasi-perpendicular angle with respect to the laser improves the electron beam generation. Shock angle deviations of even $5^{\circ }$ have noticeable effects on the beam parameters and even stability. The existence of an angle in the vertical density distribution causes an important asymmetry on the wakefield in the sudden density drop of the shock producing a potential increase on beam divergence and pointing. For cases where the gas density is sufficiently low, the vertically (gas flow direction) asymmetric expansion of the wakefield during the shock down-ramp may cause an improper injection of electrons at the back of the wakefield forming transversely oscillating secondary beams (Fig. 3). An oscillating secondary electron beam might go out with a large angle with respect to the laser axis^23^. With enough charge, such beam is capable of triggering a beam driven wakefield (Fig. 3a, green dotted square). Such secondary beams can be observed experimentally on the beam monitor as seen in Fig. 1. Though fairly important, such gas distributions imperfections are quite ignored on LWFA simulations (Fig. 3b), where it is common to consider that the distribution only varies on the longitudinal direction.

Tailoring the density distribution can result on changes in the wakefield evolution, improving the injection and acceleration. In a similar fashion, shaping purposefully the laser intensity distribution could be used to improve the LWFA process, or at least overcome unstable gas distribution. Using carefully aberrated beams made in a controlled way could counter the asymmetries found on the gas distribution and even amend some of the non-linear effects that occur on the case of an imperfect gas distribution for a Gaussian beam. Again, simulations often ignore the laser imperfections, even though their effect can be dramatic for LWFA.

The simulation presented in Fig. 3 were performed with the 2D version of the Particle in Cell (PIC) code FPlaser^24^, a resolution of 10,000 $\times$ 2200, cells, a window of 250 $\times$ 180 $\upmu m$ and 4 particles per cell. A 2D simulation is enough to catch the physics behind the shock angle density asymmetry, as in the horizontal plane the plasma density variations occur on a mm scale, thus, for a  25 $\upmu m$ laser is basically constant. For an experimental density distribution similar to the one in Fig. 3b,c and a Gaussian laser pulse of 23 fs and 0.7 J one can clearly observe the generation of a secondary beam as previously described (Fig. 1). Therefore, there are possibilities for the laser pulse shaping to compensate some effects due to gas imperfections and it also presents potential to improve the coupling between the laser and the wakefield during the LWFA process.

In this work 2D simulation has been used but for a complete picture 3D PIC simulations are needed. Our FPlaser 3D code^25^ is currently being modified to ensure that one would be able to properly simulate the real laser aberrations and its non-linearity during propagation inside a realistic gas distribution, therefore, allowing to perform a full simulation study of such detailed dynamics and give a general direction on the laser pulse tuning. Even though such work would help to go further into details in the understanding of the observed phenomena, it is out of the scope of this paper and will be given a dedicated publication in the near future.

### Electron beam transverse distribution

**Figure 4:**
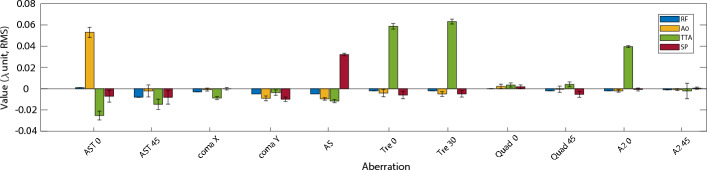
Values of the aberration and standard deviation (error bars) introduced for the configurations: reference (RF), astigmatism 0 (A0), spherical aberration (SP) and a combination of trefoil 0, 30 and second order astigmatism 0 (TTA).

A crucial parameter for any electron beam source is the transverse divergence as it has a great effect on the transport lines aperture and also on the emittance evolution^26,27^. The beam monitor measurement allows for an accurate measurement of the horizontal and vertical divergence. For the actual experiment 23 different configurations of aberrated laser pulse were tested (Supplementary Material 1). To observe the changes caused by the aberrations a “stable” and not highly sensitive configuration is needed as a reference. For that purpose, a configuration that does not trigger shock injection has been chosen. This is because a proper shock injection configuration can be highly sensitive to changes, therefore, making it difficult to gauge small variations in the electrons as it gives a more binary reaction (proper shock injection or not). However, a configuration giving a larger spectrum of energy and with enough stability in pointing, size and energy allows the observations of subtle change. The used reference is shown in Fig. 4, where all aberrations are well compensated.

**Figure 5:**
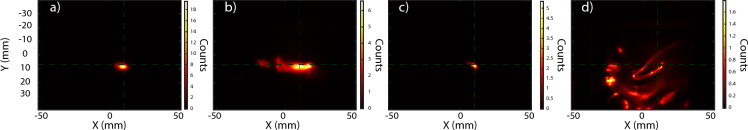
Sum of a set of consecutive shots normalized by each max intensity on the beam monitor for the aberration configurations (**a**) reference (RF), (**b**) first order astigmatism 0 (A0), (**c**) a combination of trefoil 0, 30 and second order astigmatism 0 (TTA), and (**d**) spherical aberration (SP).

**Figure 6:**
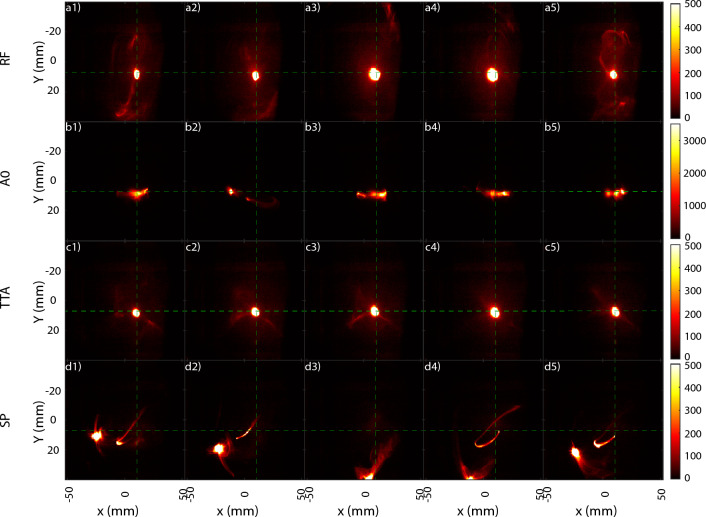
Five consecutive images of the electron beam transverse distribution recorded in the beam monitor for the aberration configurations (**a**) reference (RF), (**b**) first order astigmatism 0 (A0), (**c**) a combination of trefoil 0, 30 and second order astigmatism 0 (TTA), and (**d**) spherical aberration (SP). Background treated by the erasure of an average background noise and the use of a median filter..

To illustrate the reference configuration a selection of 3 sets of 20, 10 and 10 shots taken in the same day with a temporal separation of around a couple of hours have been selected. Taking into account all sets shots and making an intensity distribution map on the BM screen (Fig. 5) by normalizing each shot one can see the transverse shot position probability during a long experiment. Fig. 6a presents five consecutive shots of the reference configuration. An upper and lower pattern can be observed in all shots, but more strongly in Fig. 6a5. This pattern can be caused by secondary wake injections and also by electrons improperly injected in the main wakefield like in the case of an asymmetric shock (Fig. 3b). The reference configuration shows a vertical (horizontal) pointing stability of less than 0.75 mrad (around 2 mrad). The mean beam position with respect to the laser axis is of 1.2 mm in both directions. The mean total beam divergence found is 1.1 mrad (0.86 mrad) in the horizontal (vertical) direction in RMS. Though many aberrations configurations were tested (Supplementary Material 1), for clarity, in this section four of them are presented. Following the Zernike polynomials nomenclature^28–30^, the configurations are the following, i.e., reference (RF), astigmatism 0 (A0), spherical aberration (SA) and a combination of trefoil 0, 30 and second order astigmatism 0 (TTA) (Fig. 4).

When adding an horizontal first order astigmatism phase to the laser (with a RMS value of $\lambda$/20, see Fig. 4) a clear change on the electron beam is observed. Instead of a single beam (Fig. 6a), with some weak pattern around, three beams separated by up to 10 mm in the horizontal direction and quasi aligned in the vertical appear (Figs. 5b, 6b) are observed. The three beams and the horizontal separation can be linked to the laser transverse distribution evolution in this configuration. For a laser with first order astigmatism 0, before and after the focus position the laser shows a double peak around the center structure in the horizontal axis^11^, which for a sufficiently intense laser can cause multiple wakefield or a separation of the main wakefield and thus, the observed multiple beams. Considering in each shot the most intense of the three beams as the center (Fig. 6b), one finds a much worse pointing, horizontal size and divergence (Table 1). The variation of the total counts on the beam monitor ($C_{total,std}$) can be used as an approximate diagnostic of the shot-to-shot relative charge variation. The A0 configuration doubles the RF $C_{total,std}$ value.

Using a configuration with 0.03 $\lambda$ spherical aberration creates an unusual pattern on the transverse direction. The pattern presents two distinctive zones, i.e., a hook shaped beam starting from the laser axis and a secondary more circular beam always situated on a fourth of a circle on the bottom left quadrant of radius $\approx 30$ mm (Fig. 6d). The summary of a 20 shots set (Fig. 5d) confirms the consistent positioning of the hook and the forth circle. This can be caused by the effect of both the laser aberrated wavefront distribution and the shock asymmetry giving a bottom left push to the electron beams causing a secondary beam (Fig. 3) to go on the same general direction while the properly injected beam inside the wakefield starts to oscillate, thus, causing the hook shape with an end close to the center. Fourier propagation calculations on vacuum show that the laser wavefront intensity distribution develops an intense ring structure before focus that is then perturbed by a sudden asymmetric density decrease. Such structure can cause oscillations of the wakefield and even facilitate the escape of a secondary electron beam (Fig. 3) that end up developing such electron beam transverse distribution. Regarding the beam parameters, as the center or main beam are not clear to define, no position values are shown (Table 1).

Until this point, the clear effect of the addition of individual aberrations has been unequivocally shown and also that some aberrations can cause quite unique electron beam transverse patterns by which they can even be identified. However, A0 and SP are a downgrade in terms of beam parameters and stability. When adding only both trefoil together and only second order astigmatism 0 (Supplementary Material 1) improvements over the RF case were observed. Therefore, the TTA configuration was conceived with a combination of 0.06 $\lambda$ of both trefoil 0 and 30 and 0.04 $\lambda$ second order astigmatism 0 (Fig. 4). Shot-to-shot the TTA configuration presents a cleaner and more stable background compared to any of the other configurations (Fig. 6c). Comparing the total background (pixels with an intensity above 1% from the beam maximum one) signal of RF and TTA (where one can clearly define a main beam) gives that RF has in average 1.5 times more background signal than the TTA cases with a shot-to-shot std of 8.9% for RF and 11% for TTA with respect to their mean value. The hit zone of 20 consecutive shots is smaller (Fig. 5c) than the RF (Fig. 5a) and only 2 shots impinge around 1.5 mm to the left of the beam monitor center. All measured parameters see an improvement in both stability and mean as seen in Table 1, specially the transverse divergence and beam size where the standard deviation is reduced by nearly an order of magnitude and the mean by more than half when compared to the RF. $C_{total,std}$ doesn’t suffer much change and due to the already approximate character of the measurement it is considered equal to the RF case. The specific dynamics of the laser and subsequent wakefield in the TTA configurations are quite complex and require of a more individual study considering 3D effects to understand in which cases it will produce an improved as the observed one. Nevertheless, there are clear signs that one can achieve improvements on the beam parameters by tuning the wavefront via the controlled introduction of aberrations to compensate for the possible asymmetries on the gas shock.

**Table 1:** Mean and standard deviation transverse position, divergence, size and relative total count standard deviation measured at the beam monitor of the configurations: reference (RF), astigmatism 0 (A0), spherical aberration (SP) and a combination of trefoil 0, 30 and second order astigmatism 0 (TTA).

Config	Position X (mm) [std]	Position Y (mm) [std]	$\sigma '_{x,RMS}$ (mrad) [std]	$\sigma '_{y,RMS}$ (mrad) [std]	$\sigma _{x,RMS}$ (mm) [std]	$\sigma _{y,RMS}$ (mm) [std]	$C_{total, std}$ ($\%$)	$\#$ shots
RF	− 1.05 [2.03]	1.31 [0.76]	1.14 [0.4]	0.89 [0.18]	1.08 [0.38]	0.85 [0.17]	17	45
A0	− 5.57 [13.01]	0.25 [2.02]	1.85 [1.11]	0.9 [0.33]	1.46 [1.06]	0.86 [0.32]	38.53	20
SP	–	–	1.06 [0.6]	0.69 [0.28]	1.01 [0.56]	0.66 [0.27]	27.78	20
TTA	− 0.76 [1.87]	0.46 [1.11]	0.43 [0.07]	0.50 [0.06]	0.41 [0.067]	0.48 [0.06]	17.51	20

### Electron beam energy distribution

The energy distribution and charge are crucial parameters for a lot of electron beam uses, e.g., FEL. The advantage of the shock injection scheme is the capability to produce low energy spread electron beams that go through a similar acceleration shot-to-shot. Therefore, there is a difference in the acceleration of the electrons between an ideal wakefield and a perturbed one due, for example, to oscillations during the shock asymmetries or to the effect of aberrated laser propagation inside the plasma.

**Figure 7:**
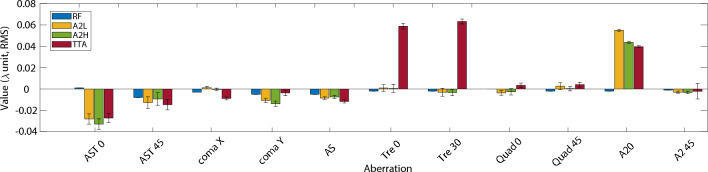
Values of the aberration and standard deviation (error bars) introduced for the configurations: reference (RF), second order astigmatism 0 low (A2L), second order astigmatism 0 high (A2H) and a combination of trefoil 0, 30 and second order astigmatism 0 (TTA).

**Figure 8:**
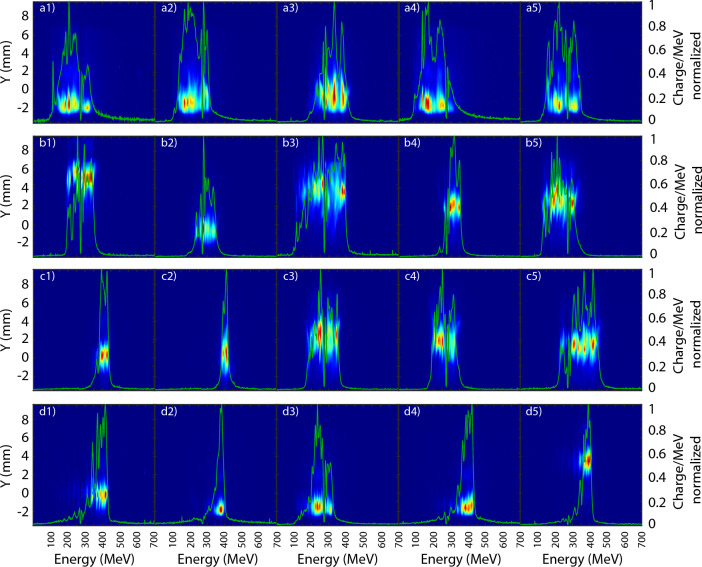
Five consecutive measurements of the normalized electron beam charge distribution recorded on the electron spectrometer $\approx\, 2$ m after the gas jet for the aberration (**a**) configurations reference (RF), (**b**) second order astigmatism 0 low (A2L), (**c**) second order astigmatism 0 high (A2H) and (**d**) a combination of trefoil 0, 30 and second order astigmatism 0 (TTA).

As previously mentioned the RF configuration (Fig. 7) does not trigger a proper shock injection. An irregular injection is triggered, leading to a large energy spread (Fig. 8a). The addition of 0.04 $\lambda$ second order astigmatism 0 (A2L) to the RF configuration Increases substantially the peak energy ($E_{peak}$) and its 1 MeV slice charge ($Q_{peak}$) while reducing the energy spread by $\approx$ 30% (Table 2). The electron beam vertical pointing becomes less stable shot-to-shot, with jumps of ±3 mm, i.e., $\approx \,1.2$ mrad pointing (Fig. 8). Further increasing the second order astigmatism 0 to 0.05 $\lambda$ (A2H) causes a significant increase in $E_{peak}$ achieving up to $\approx$ 410 MeV, while the total charge is reduced, a usual behaviour between injected charge and acceleration efficiency in shock-injection^31^.. The rest of the parameters (Table 2) also exhibits an improvement, furthermore, some shots trigger a proper shock injection (Fig. 8c1,c2), consequence of only tuning the wavefront intensity distribution and not touching any other setup parameters. The comparison between A2L and A2H makes clear the high non-linearity of the sensitivity of the LWFA to the addition of aberrations, as the change from RF to A2L is less important to most parameters than the jump from A2L to A2H (Table 2), specially for $Q_T$ and $E_{peak}$.

The TTA configuration presented a global improvement on the beam monitor (Table 1) and the same occurs on the electron spectrometer (ESM) where both means and standard deviations are better than the RF, A2L and A2H configurations (Table 2) for all parameters except $Q_T$. As in the A2H case (Fig. 8c), the TTA configuration also starts to trigger proper localized injection (Fig. 8d), however, with a 20% success rate instead of the 10% of A2H, and a slight increase in acceleration of the electrons.

By slightly moving the focus position for the A20H and TTA configurations a position with a higher rate of triggering proper shock injection was searched to compare their performance with the used gas target. Both configurations could achieve a higher success rate in generating low energy spread electron beams with $\approx$ 75% and $\approx$ 90% for A20H and TTA respectively. Both generally improve the electron beam generation with respect to their initial focus position especially in terms of $E_{peak}$ and $\sigma _{\gamma }$. As the injection is more precisely localized, $Q_T$ is lower (around half) but the smaller $\sigma _{\gamma }$ guaranties helps to avoid a drastic drop in slice charge, crucial for some applications (2). The TTA configuration achieves a higher peak energy of close to two times the one of the RF configuration with a $\approx$ 9% stability, percent level spread and under 0.5 mrad divergence, much better than the RF even when optimizing its focus. The charge values for TTA are coherent with the electron energy obtained and the laser parameters of our system (0.7 J, 25 fs, 25 $\upmu m$, on-target).

**Table 2:** Mean and standard deviation peak energy ($E_{peak}$), energy spread ($\sigma _{\gamma ,RMS}$), beam divergence ($\sigma '_{y,RMS}$), 1 MeV peak energy slice divergence ($\sigma '_{y,peak,RMS}$), peak energy 1 MeV slice charge ($Q_{peak}$) and total charge ($Q_T$) for the aberrations configuration RF, A2L, A2H and TTA.

Config	$Q_{T}$ (pC) [std]	$Q_{peak}$ (pC/MeV) [std]	$\sigma _{\gamma ,RMS}$ (MeV) [std]	$\sigma _{\gamma ,RMS}$ ($\%$) [std]	$\sigma '_{y,E_{peak},RMS}$ (mrad) [std]	$\sigma '_{y,RMS}$ (mrad) [std]	$E_{peak}$ (MeV) [std]	$\#$ shots
RF	52.20 [18.14]	0.35 [0.14]	81.73 [14.37]	37.58 [12.70]	0.53 [0.24]	0.54 [0.19]	233.23 [56.8]	20
A20L	55.49 [12.44]	0.54 [0.16]	57.23 [24.81]	22.41 [11.32]	0.63 [0.19]	0.63 [0.21]	273.08 [60.94]	10
A20H	46.29 [16.77]	0.46 [0.13]	48.74 [21.33]	14.88 [7.25]	0.54 [0.20]	0.57 [0.11]	349.52 [72.67]	20
TTA	34.44 [10.36]	0.40 [0.09]	31.33 [10.98]	9.30 [4.36]	0.38 [0.09]	0.40 [0.09]	355.81 [54.81]	10
A20H (F)	17.17 [7.78]	0.3 [0.24]	15.65 [5.95]	4.42 [1.74]	0.27 [0.12]	0.43 [0.39]	361.53 [68.35]	20
TTA (F)	18.19 [7.85]	0.29 [0.15]	15.96 [5.91]	3.74 [1.44]	0.31 [0.05]	0.45 [0.12]	428.32 [41.4]	10

## Discussion

Previous recent works and 2D PIC simulations here presented confirm the crucial effect of asymmetries in the gas distribution seen by the laser and their great consequences on the intra-wakefield electron beam dynamics causing from higher pointing and shot-to-shot instabilities to even the generation of multiple electron beams due to PWFA sparking from the oscillating main electron beam. In this work we confirm experimentally and with PIC simulations how the tuning of the laser pulse wavefront via the controlled addition of aberrations can be used to manipulate the laser propagation, so, the generated wakefield can compensate the gas target irregularities and provide better electron beam parameters and even stability. In addition, clear reproducible unique transverse beam distributions have been observed that further show the large difference of the LWFA process for each laser configuration. Experimentally, the wavefront manipulation has allowed to reduce the pointing, increase the peak energy by close to two times while reducing the divergence and attain proper shock injection. All of this was achieved using a wavefront sensor and a deformable mirror, both of which are commonly used in LWFA facilities, thus, the implementation of this technique is quite simple but can potentially improve laser–plasma coupling. However, as it has been demonstrated that the aberrations effect is not linear, it remains to be seen whether if the same aberrations configuration here applied for the improvements is applicable in general or they should be modified taking into account factors like gas composition or gas length. Our results show clearly that our optimum wavefront intensity distribution is actually quite far from a case without aberrations. It may also be possible to find a laser configuration superior to the Gaussian case even in ideal gas distribution conditions. A future work exploring in-depth the details of the laser propagation during LWFA is underway to answer such questions. Another possible use of this technique is the generation of multiple well separated different beams, that could be guided through magnetic elements to have two synchronized electron beams from a single LWFA system. Finally, once better understood a facility with proper real-time wavefront and gas distribution diagnostics could make use of machine learning methods to delegate the complexity of selecting a proper transverse density distribution for various purposes to a neural network.

## Methods

### Laser system

The laser system at LAPLACIAN facility, where experiments were conducted, was designed and constructed by Amplitude (Evry, France). It is a 40-TW Ti:Sa laser system (with a broadband spectrum from 750 nm to 850 nm), which delivers up to 0.7 J on-target with a pulse duration of about 23 fs (FWHM). The 80 mm initial beam is focused by an off-axis parabola (OAP) working with a f-number of 20 resulting in a Gaussian-like focal spot with a waist diameter of 20 $\upmu m$. The laser system can be operated up to 5 Hz. To improve the beam contrast ratio, a cross-polarization wave technique is used in the front-end part; an AOPDF (Acousto-Optic Programmable Dispersive Filter, DAZZLER) is inserted just after the stretcher to manipulate the spectral phase to improve the beam compression quality; an AOPGCF (Acousto-Optic Programmable Gain Control Filter, MAZZLER) is also implemented inside the regenerative amplifier to thwart gain narrowing issues and broaden the amplified pulse bandwidth (from 35 to 100 nm), which enables shorter pulse duration, after compression.

### Deformable mirror and wavefront measurement

A wavefront sensor (SID4 from Phasics with 182x136 sampling points over a 5 $\times$ 3.6 mm^2^ aperture) is used to collect a leakage measurement before the compressor, and an adaptive optics loop through a large-size deformable mirror (5$^{\prime \prime }$, with 35 actuators) is run. Any given phase map can be described using the decomposition with Zernike Polynomials which is a family of polynomials that are orthogonal to each others on a unit disk so that the phase can be expressed as a linear combination of different polynomials. For example, the second and third Zernike polynomials corresponds to the tilt (0 and 45 degres) while the 8th and 9th corresponds to vertical and horizontal coma. Then, this phase can be sent as a target during the correction loop, which enables to shape the beam and introduce aberrations.

Another wavefront sensor (same reference) has also been used after the OAP with a diverging lens to make the incident beam collimated and small enough to fit the aperture size of the sensor. We ensured that the laser beam line was correctly aligned, especially the OAP, and that no extra aberrations were added before sending the laser to the gas target.

### Gas target

The supersonic gas jet used is a simple conical nozzle of 3 mm length and mach $\approx \,3.8$ for hydrogen provided by QST. The jet is mounted on a motorized stage that offers 3 degrees of freedom for movement. The used blade is made of stainless steel and with a thinness of 10 $\upmu m$ at the edge. The supersonic gas hitting the blade could cause some vibration. in order to reduce the vibration the blade is attached by two stainless steel structures close to parallel in the longitudinal direction. The blade is mounted on a motorized stage that offers 2 degrees of freedom for movement. The gas distribution has been measured using a Mach–Zehnder interferometer and a gas vibrations of $\approx \,10$ $\upmu m$ has been measured.

### Electron beam diagnostics

A beam monitor is used to image the transverse distribution of the electron beam during experiment. The beam monitor is a DRZ of 15 cm by 15 cm total size. the monitor is positioned at   955 mm from the gas jet with a 45 degrees angle back imaged by a BU-51LN camera. An aluminium foil of 100 $\upmu m$ thickness is positioned in front to filter some laser and plasma radiations. The electron energy spectrum is measured in an ESM. The ESM is composed of 2 permanent magnet dipoles of 0.55 T on a movable stage to take it out of the laser axis for further transport, a 0.62 m long DRZ-HIGH phosphor screen and two EMCCD Pro-HS1KBX3 cameras to image the 0–260 MeV range and 230–800 MeV range of the screen. The ESM entrance is positioned $\approx \,2$ m after the gas jet and a $\approx \,12$ mm ($\approx \,40$ mm) vertical (horizontal) aperture. The screen is positioned with an angle of 45 degrees and top imaged by the two cameras.

### Particle in cell code

The 2D version of the fully relativistic FPLaser code was used for all simulations here shown. The simulations were done with a “moving window” moving at the speed of light of 250 $\upmu m$ longitudinal and 180 $\upmu m$ transverse size, 10,000 by 2200 cells and 4 particles per cell. The simulations were done in the HPE SGI8600 supercomputer using 200 CPU cores.

### Supplementary Information

Supplementary Information.

## Data Availability

The datasets used and/or analysed during the current study available from the corresponding author on reasonable request.
