# Supplementary material for: Notable improvements on LWFA through precise laser wavefront tuning
Source: Sci Rep. 2023 Oct 27;13:18466. doi: 10.1038/s41598-023-45737-5 (PMC10611724; doi:10.1038/s41598-023-45737-5)
Supplement: Supplementary file 1 — Supplementary Information. [file 41598_2023_45737_MOESM1_ESM.pdf]

1 Supplementary material: Tested aberration configurations

Table 1 Summary of aberration configurations experimentally tested. For a given configuration, the changed family of aberration value is shown in blue. The coefficient a_1, a_2, ..., a_{12} stand for defocus, astigmatism 0 °and 45 °, vertical coma, horizontal coma, spherical aberration, trefoil 0 °, trefoil 30 °, quadrafoil 0 °and 25 °, and second order astigmatism 0 °and 45 °. All the aberration coefficients presented in this table were averaged over 10 to 50 values depending on the number of shot conducted for each configurations.

**Configuration**	**Aberrations (λ unit)**											
Name	a_1	a_2	a_3	a_4	a_5	a_6	a_7	a_8	a_9	a_{10}	a_{11}	a_{12}
C-H-1	-0.13	-0.013	-0.0018	0.058	-0.0094	-0.0058	-0.0071	-0.0069	0.0024	-0.0008	0.0017	-0.0044
C-H-2	-0.13	-0.0078	-0.0045	0.044	-0.0087	-0.0067	-0.0065	-0.0049	0.0007	-0.0003	0.0005	-0.0041
C-HV-1	-0.14	-0.0061	-0.001	0.059	0.051	-0.0074	-0.0089	-0.0052	0.0027	-0.0008	0.0016	-0.0026
C-HV-2	-0.14	-0.0017	-0.006	0.045	0.038	-0.0078	-0.0090	-0.0042	0.0023	-0.0016	0.0003	-0.002
D	-0.05	-0.012	-0.0009	-0.0011	-0.01	-0.0097	-0.0054	-0.005	0.0023	0.0008	-0.0006	-0.0011
SA-1	-0.17	-0.008	-0.01	0.003	-0.012	0.047	-0.0072	-0.003	0.00025	0.007	-0.0016	-0.0009
SA-2	-0.17	-0.007	-0.009	0	-0.01	0.032	-0.006	-0.0049	0.0018	-0.0053	-0.0007	-0.0005
A0H	-0.096	0.079	-0.0025	-0.0013	-0.011	-0.0091	-0.0019	-0.049	0.0020	-0.0002	-0.0025	-0.0016
A0M	-0.09	0.053	-0.0021	-0.0009	-0.009	-0.0096	-0.0041	-0.005	0.0022	-0.0005	-0.0022	-0.0008
A0	-0.1	0.038	-0.0043	-0.0014	-0.012	-0.0089	-0.0017	-0.0043	0.0029	-0.0006	-0.0011	-0.0016
A-045-1	-0.11	0.055	0.055	-0.002	-0.0088	-0.0086	-0.0011	-0.0035	0.0013	-0.0005	-0.0022	-0.0023
A-045-2	-0.19	0.035	0.021	-0.004	-0.009	-0.0069	-0.0033	-0.003	0.0032	-0.0048	-0.0042	-0.0037
TTH	-0.16	-0.0092	-0.0004	-0.0023	-0.011	-0.0077	0.055	0.055	0.006	0.0012	-0.0005	-0.001
TTL	-0.16	-0.0044	-0.0046	-0.0015	-0.0089	-0.0074	0.040	0.040	0.0053	0.0015	-0.0016	-0.0012
Q-1	-0.18	-0.0079	-0.037	-0.0042	-0.01	-0.0096	-0.0066	0.0022	0.043	0.036	0.0012	-0.0025
Q-2	-0.17	-0.0054	-0.014	-0.0021	-0.012	-0.012	-0.0052	0.0039	0.054	0.049	0.0008	-0.0025
A2-0-1	-0.0067	-0.049	-0.0061	-0.0095	-0.0026	-0.015	-0.0063	0.004	0.0049	0.0067	0.081	-0.0005
A2-0-2	-0.17	-0.028	-0.013	-0.0012	-0.011	-0.0086	0.0009	-0.003	-0.0037	0.0028	0.055	-0.0029
A2-0-3	-0.18	-0.033	-0.0093	-0.00037	-0.014	-0.0075	0.0005	-0.003	-0.0026	0.0004	0.044	-0.0033
A2-045-1	-0.17	-0.03	-0.037	0.001	-0.0071	-0.01	0.0057	0.0016	-0.0058	0.0096	0.052	0.051
A2-045-2	-0.2	-0.03	-0.02	0.001	-0.001	-0.0065	0.0026	-0.0022	-0.0037	0.0037	0.041	0.037
TTA	-0.06	-0.032	-0.015	-0.0088	-0.0037	-0.012	0.059	0.063	0.0035	0.0041	0.04	-0.0022

Table 1 presents all the aberration configurations done during the experiment with their correspondent shot-to-shot variation in λ units. To avoid over-complicating the results shown, not all tested configurations will be shown here. In Fig. 1 five consecutive shots on the beam monitor for five additional configurations are shown. Fig. 1a,b together with Fig. 6b (main manuscript), exhibit the sensitivity of the LWFA process to the astigmatism 0. Adding a 25 % more astigmatism 0 with respect to the A0 configuration (Table 1) makes the shape less stable in comparison and starts to display a multiple beam cluster to the around 25 mm to the left of the laser axis. Further increasing by 0.03 λ, vastly improves the apparition rate of the left cluster shape with the smudge on the right (Fig. 1b). The introduction of a 0.04 of second order astigmatism 0, creates on the beam monitor a faint circular pattern of radious $\approx$25 mm around a centered main beam (Fig. 1d). In some occasions (10 % of the shots) a secondary far beam appears on top of the circle. The configuration A2-045-1 is composed of 0.03 first order astigmatism 0 and 45 and 0.05 second order astigmatism 0 and 45 (Table 1, Fig. 1c). 65 % (15 %) of the shots presents a nice beam shifted around 10 mm to the left to the laser axis with a weak circular pattern of 20 mm radious with a (two) bigger secondary beam on top. However 20 % of the shots something more akin to the multiple beams of the first order astigmatism without the weak circular pattern around it. Finally the ensemble of both trefoil aberrations (TTH) with a value of 0.55 (Table 1, Fig. 1d) generates a complex pattern with a beam slightly shifted

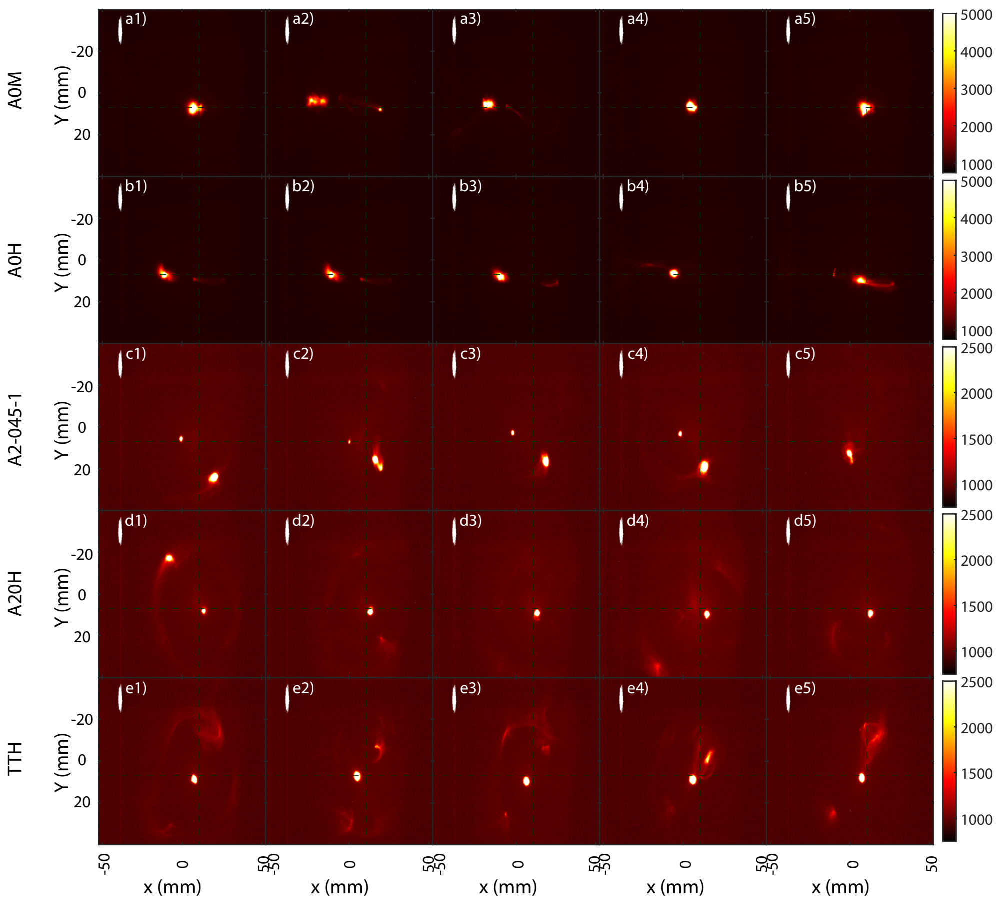

Fig. 1 Five consecutive RAW images of the electron beam transverse distribution recorded in the beam monitor for the aberration configurations a) A0M, b) A0H, c) A2-045-1, d) A20H and e) TTH (Table 1).

from the laser axis and two weak beams aligned in a diagonal line, with an apparition rate of 75 %.

2 Supplementary material: Wavefront phase maps

In Figure 2, the wavefront error map (RF - reference ; A0 - astigmatism ; SP - spherical aberration ; A20L and A20H - second order astigmatism ; TTA - trefoil and astigmatism) associated with the used experimental configurations are shown. As expected, the reference phase is close to a flat wavefront (almost no aberrations). The TTA configuration that produced the best electrons, and that is a non-perfect wavefront map, would clearly be considered as unwanted experimentally in a lot of fields in optics while for our LWFA experiments it gave rise to improvements of the beam.

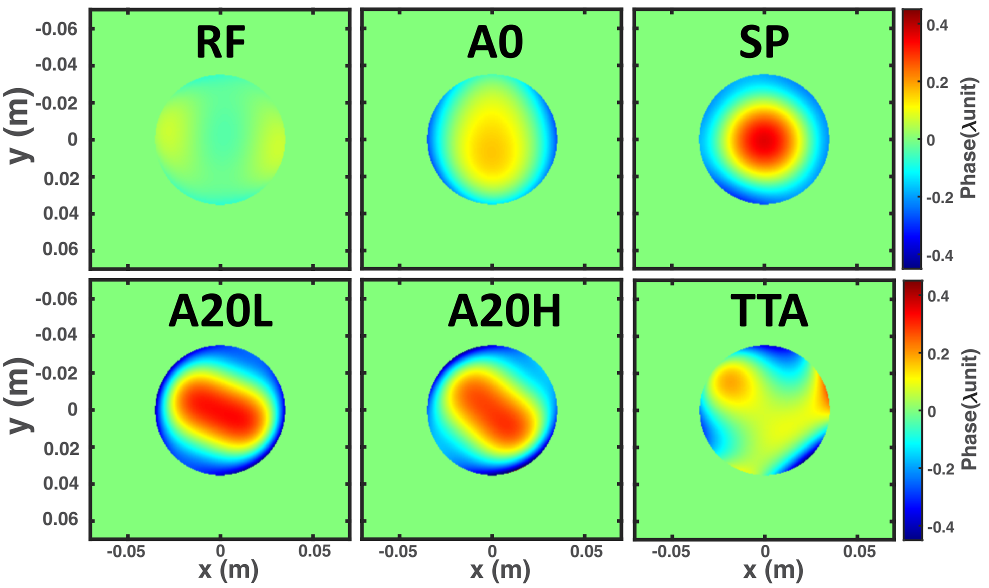

Fig. 2 Wavefront phase map for the configurations presented in Table 1 and 2. The error phase is expressed in wavelengths unit ($1\lambda = 2\pi$ radians)
